# Editorial: Advancements in the understanding, diagnosis, and control of viral diseases of cattle, goats, and sheep

**DOI:** 10.3389/fcimb.2026.1914501

**Published:** 2026-07-07

**Authors:** Wang Wei

**Affiliations:** 1 School of Life Sciences, Inner Mongolia University, Hohhot, China; 2 Lanzhou Veterinary Research Institute Chinese Academy of Agricultural Sciences, Lanzhou, China

**Keywords:** control, diagnosis, molecular surveillance, ruminants, vaccine, viral disease

Viral diseases remain among the most important constraints to the sustainable development of cattle, goat, and sheep industries worldwide. Their impact is particularly evident in respiratory disease complexes, enteric infections, reproductive disorders, transboundary animal diseases, and emerging infections in pastoral and high-altitude production systems. In addition to direct production losses, these diseases challenge disease surveillance, animal movement, vaccination strategies, and farm biosecurity. This Research Topic brings together ten contributions that collectively advance our understanding of viral diversity, molecular epidemiology, host–virus interactions, diagnostic innovation, and regional control strategies for ruminant viral diseases.

Several studies in this Research Topic highlight the value of molecular surveillance for detecting emerging or under-recognized viruses in ruminant populations. Pan et al. identified a novel Ungulate copiparvovirus 10 in sheep from Hami, East Xinjiang, China, based on virome analysis and molecular characterization. This work expands current knowledge of copiparvovirus diversity in small ruminants and emphasizes the importance of monitoring border and pastoral regions where livestock movement, wildlife interfaces, and ecological factors may facilitate viral emergence. Similarly, Chen et al. reported the detection and molecular characterization of bovine enterovirus E2 from dairy calves with respiratory disease in Urumqi, Xinjiang, China. Their findings suggest that bovine enterovirus may be involved in calf respiratory disease and support the inclusion of less routinely investigated viruses in respiratory disease surveillance programs.

Bovine viral diarrhea virus was a major focus of this Research Topic, reflecting its continuing importance in cattle and yak production. Li et al. reviewed the current epidemic status, prevention, and control of bovine viral diarrhea virus in yaks in China, proposing an integrated framework that combines vaccine improvement, precise monitoring, dynamic early warning, population purification, and active prevention. Complementing this review, another study by Li et al. conducted a comprehensive molecular epidemiological investigation of bovine viral diarrhea virus in yaks in Qinghai Province and reported high seroprevalence, active infection, and the predominance of BVDV-1u. These findings provide important evidence that bovine viral diarrhea virus is widely circulating in yak populations and that prevention strategies for high-altitude pastoral systems should be tailored to local epidemiological and management conditions. Wang et al. further contributed to bovine viral diarrhea control by expressing the E2 protein in CHO-S cells and developing an indirect ELISA for serological detection. This method provides a useful tool for antibody monitoring, herd surveillance, and vaccine efficacy evaluation.

Respiratory viral disease remains a central challenge in cattle health management. Ban et al. developed and evaluated a multiplex RT-qPCR assay targeting the F and N genes of bovine respiratory syncytial virus and analyzed viral load in cattle with bovine respiratory disease complex in Inner Mongolia, China. Their data revealed substantial detection of bovine respiratory syncytial virus among clinically affected calves and showed differences associated with sample type, region, season, and farm management system. These findings provide baseline molecular epidemiological information for bovine respiratory syncytial virus in one of China’s major cattle-producing regions and support the application of molecular diagnostics for targeted monitoring and outbreak investigation. At the level of host–virus interaction, Zhu et al. demonstrated that bta-miR-146b promotes infectious bovine rhinotracheitis virus replication by targeting IRAK1 and inhibiting type I interferon expression. This study provides mechanistic insight into how host microRNAs may modulate antiviral immunity and viral replication during infection.

Diagnostic innovation was another important theme of this Research Topic. Wang et al. developed an RPA-CRISPR/Cas12a assay for rapid on-site detection of major calf diarrhea pathogens, including bovine viral diarrhea virus, bovine coronavirus, bovine rotavirus, and enterotoxigenic Escherichia coli. The assay can be completed rapidly in a closed-tube format and showed high sensitivity and specificity. Such field-deployable diagnostic platforms are particularly valuable for early decision-making in farms where timely pathogen identification is essential for reducing calf morbidity, mortality, and unnecessary antimicrobial use.

The Research Topic also addresses viral diseases with clear transboundary or vector-borne significance. Bora and Karki discussed host expansion and interspecies infection of peste des petits ruminants virus in the context of global control and eradication. Their article emphasizes that successful peste des petits ruminants control requires a deeper understanding of host susceptibility, wildlife–livestock interfaces, ecological drivers, and transmission dynamics beyond traditional domestic small ruminant hosts. Wang et al. reviewed the epidemiology and regional control strategies of Schmallenberg virus, with emphasis on diagnostics, vaccine development, and Culicoides vector management. Their review highlights that climate change, animal trade, and vector ecology can reshape the risk landscape for ruminant viral diseases and that control strategies must be adapted to both endemic and non-endemic settings.

Taken together, the articles in this Research Topic demonstrate that progress in controlling viral diseases of cattle, goats, and sheep requires an integrated approach. Molecular epidemiology and metagenomics can reveal viral diversity and emerging pathogens; mechanistic studies can uncover host factors and immune pathways that influence infection; improved serological and nucleic-acid-based diagnostics can support surveillance and rapid field response; and region-specific prevention strategies can translate scientific findings into practical disease control.

Looking forward, although viral pathogens play a crucial role in many major diseases of ruminants, they should not be considered in isolation. Under the global demand for antimicrobial stewardship, reduced antibiotic use, and the development of alternatives to antibiotics, bacterial diseases are becoming increasingly important in ruminant health management. Their prevention and control can no longer rely primarily on therapeutic antimicrobial intervention, but should instead be addressed through early diagnosis, vaccination, biosecurity, microbiome regulation, and integrated herd health management. Therefore, future Research Topics may place greater emphasis on the epidemiology, diagnosis, immune prevention, and vaccine development of bacterial diseases in cattle, goats, and sheep. In particular, comprehensive studies on respiratory and digestive disease complexes, in which viral, bacterial, environmental, nutritional, and management factors interact, will be essential. Only through systematic and integrated prevention and control strategies can we effectively safeguard the health, productivity, and sustainable development of the ruminant industry.

